# Improving childhood vaccination in minorities: a realist review of health system interventions: Jumanah Essa-Hadad

**DOI:** 10.1093/eurpub/ckac131.395

**Published:** 2022-10-25

**Authors:** J Essa-Hadad, Y Gorelik, M Edelstein

**Affiliations:** Faculty of Medicine, Bar-Ilan University, Safed, Israel

## Abstract

**Background:**

Most minority populations in Europe have lower childhood vaccine uptake than the general population. Improving uptake in these populations requires specifically developed, context-specific interventions. As part of the EU-funded RIVER-EU project, we conducted a realist review to identify interventions effective at improving vaccine uptake among underserved communities.

**Methods:**

We searched MEDLINE, EMBASE, CINAHL, COCHRANE, and Proquest for articles published between 2005 and 2022, using combination keyword searches in English. Following title and abstract screening, full texts were assessed for relevance. We also searched grey literature and references of references. Data extraction and analysis was performed by two reviewers. Programme theories were generated from included articles and data extraction were carried out paying particular attention to context, mechanisms, and outcome configurations.

**Results:**

From 1942 screened titles we selected 87 studies for full-text review of which 34 were included. We identified 10 primary intervention categories: parental and youth education; clinic outreach; quality improvement; health provider training; school-based education; technology interventions; cash incentives; home visits; comic books; community leaders education; and consent policy changes. The analysis highlighting contextual factors enabling or hindering each intervention category’s success is ongoing.

**Conclusions:**

Several intervention categories can potentially improve vaccine coverage among underserved minority populations. We will describe their effectiveness and the contextual factors contributing to their success or failure to inform the development of tailored interventions targeting these populations.

**Key messages:**

• Improving vaccination in underserved minority populations requires identification of effective interventions, barriers/enablers to their success, specific to the context in which they are implemented.

• Appropriately tailored health system interventions are effective at improving vaccine uptake among underserved minority communities.

